# Effect of a water-maze procedure on the redox mechanisms in brain parts of aged rats

**DOI:** 10.3389/fnagi.2015.00029

**Published:** 2015-03-11

**Authors:** Natalia A. Krivova, Olga B. Zaeva, Valery A. Grigorieva

**Affiliations:** Laboratory of Experimental Physiology, Institute of Biology and Biophysics, Tomsk State UniversityTomsk, Russia

**Keywords:** aging, brain, water-maze procedure, oxidants, antioxidants

## Abstract

The Morris water maze (MWM) is a tool for assessment of age-related modulations spatial learning and memory in laboratory rats. In our work was investigated the age-related decline of MWM performance in 11-month-old rats and the effect exerted by training in the MWM on the redox mechanisms in rat brain parts. Young adult (3-month-old) and aged (11-month-old) male rats were trained in the MWM. Intact animals of the corresponding age were used as the reference groups. The level of pro- and antioxidant capacity in brain tissue homogenates was assessed using the chemiluminescence method. A reduced performance in the MWM test was found in 11-month-old rats: at the first day of training they showed only 30% of successful MWM trials. However, at the last training day the percentage of successful trials was equal for young adult and aged animals. This indicates that the aged 11-month-old rats can successfully learn in MWM. Therewith, the MWM spatial learning procedure itself produces changes in different processes of redox homeostasis in 11-month-old and 3-month-old rats as compared to intact animals. Young adult rats showed a decrease in prooxidant capacity in all brain parts, while 11-month-old rats demonstrated an increase in antioxidant capacity in the olfactory bulb, pons + medulla oblongata and frontal lobe cortex. Hence, the MWM procedure activates the mechanisms that restrict the oxidative stress in brain parts. The obtained results may be an argument for further development of the animal training procedures aimed to activate the mechanisms that can prevent the age-related deterioration of performance in the learning test. This may be useful not only for the development of training procedures applicable to human patients with age-related cognitive impairments, but also for their rehabilitation.

## Introduction

The Morris water maze (MWM) (Morris, 1984) or the spatial water maze is a classical test for assessment of spatial learning and memory, which is often used for appraisal of cognitive deficits upon aging. Rodents (rats or mice) are employed as model animals in this test to explore the mechanisms of age-related cognitive decline and form a basis for rehabilitation. The development of cognitive deficits in rodents is related to physiological aging rather than to the death of brain cells (Foster, 2006). Successful spatial learning and memory are determined by the integrity and functioning of the hippocampus as well as by the involvement of adjacent regions of the temporal cortex and some structures of the temporal pole (Jarrard, 1993; Driscoll et al., 2006). It is supposed that the formation of cognitive deficits in rodents, as in humans, starts in the middle age (11–14 months) and increases during the life (Bizon et al., 2009). The early detection of risk factors of cognitive impairment may facilitate a search for new therapeutic approaches and provide healthy aging (Konishi and Bohbot, 2013; Morris et al., 2014). The use of different versions of the MWM made it possible to elucidate various physiological and cellular mechanisms of cognitive impairment in the aged brain (Murchison et al., 2009; Foster, 2012; Griffith et al., 2014).

Such experiments are important also because the animal training procedures can be used to develop the advanced virtual media technologies for treating age-related cognitive declines in human (Foster et al., 2012). However, these technologies should probably take into account the so-called carryover effects, which occur when preliminary training affects the results of further training. Guidi M. and co-authors considered the role of carryover effects in determining the age-related impairment of episodic and reference memory in two versions of the water maze task. Their study has demonstrated that preliminary Cue discrimination training and Initial spatial discrimination training produced an effect on deterioration of episodic and reference memory. It should be noted that tests of episodic spatial memory were more reliable than reference memory for detecting cognitive decline (Guidi et al., 2014). Thus, the testing procedure may be accompanied by changes in brain functioning and may have an impact on the cognitive sphere.

The occurrence of carryover effects seems to be useful for training the patients with age-related deficits. In recent years, new technologies based on Serious Games have been devised for assessment of patients with Alzheimer's disease and related disorders (ADRD). It was found that the use of Serious Games can aid not only in assessment of functional disturbances but also in treatment of ADRD (Robert et al., 2014). In particular, cognitive games can improve attention and memory. After such games, patients with mild cognitive impairment showed an improvement of memory scores along with activation of the hippocampus (Rosen et al., 2011). Physical games (Wii Sports) in Alzheimer's patients not only improved the scores but also normalized the patient's balance and gait (Legouverneur et al., 2011). Hence, the training technologies can be used as therapeutic methods. Therewith, it is necessary to elucidate functional changes that occur in brain during the training process.

Many functional changes in the brain activities are related to the progressive oxidative stress, which provokes neurodegenerative diseases (Sandhu and Kaur, 2002). In general terms, the oxidative stress is interpreted as an imbalance in prooxidant/antioxidant homeostasis, which is shifted toward prooxidants (a review by Dalle-Donne et al., 2003). Prooxidants and antioxidants represent various molecular mechanisms. The level and state of the oxidative stress depend on the activity of pro- and antioxidants and their interaction. The basis of age-related neuronal dysfunction can be revealed by the integrated determination of prooxidant and antioxidant capacity of a particular sample of brain tissue. This will outline the direction of further investigation of specific molecular targets of the oxidative stress, which could help to devise a therapy against cognitive impairment of aged brain.

Our work was aimed to reveal the effect of the water-maze procedure on the level of oxidative stress in different brain parts of aged rats. The level of oxidative stress was assessed from the ratio of pro- and antioxidant capacity in brain tissue homogenates using the chemiluminescence method. Young adult (3-month-old) and aged (11-month-old) male rats were trained in the water maze. Intact animals of the corresponding age were used as the reference groups.

## Materials and methods

### Animals and experimental design

All animal procedures were in compliance with the European Communities Council Directive of 24 November 1986 (86/609/EEC) and approved by the Animal Research Ethics Committee at the Institute of Biology and Biophysics, Tomsk State University.

Male Wistar rats, 2 and 10 months old, were purchased from a nursery at the Institute of Pharmacology, Siberian Division of the Russian Academy of Medical Sciences. The rats were kept in an isolated and ventilated room in the vivarium at the Institute of Biology and Biophysics, Tomsk State University. A temperature of 20 ± 2.0°C and air humidity of 60% were maintained in the room with a 12 h light: 12 h dark diurnal cycle. All the animals were given ad labium access to food and water (a standard rat diet). Rats were monitored during a 4 week period.

After a quarantine period, rats of each age were randomly divided into 2 groups: 3-month-old and 11-month-old rats, 10 animals in each group, thus forming 4 groups, with 2 groups of each age. Animals of the first and second groups were trained in the MWM (“trained 3-month-old rats” and “trained 11-month-old rats,” respectively). Animals of the third and fourth groups (“not trained 3-month-old rats” and “not trained 11-month-old rats,” respectively) were used as the reference. Data obtained for the trained rats were compared with the corresponding data for not trained animals of the same age.

### A water-maze procedure (MWM)

All the test animals were allowed to swim in a pool for 60 s. Training was performed by a standard procedure using a pool 1.5 m in diameter and 0.6 m in height with a hidden platform 10 cm in diameter and three different geometrical figures on the pool walls as landmarks. The arrangement of such landmarks and the starting point were always constant. Each training trial took 60 s. If the trial failed and the rat did not find the hidden platform during 60 s, the animal was placed on the platform for 10–15 s. Each rat received four training trials a day for 4 consecutive days (days 1, 2, 3, and 7). The time of successful trial for each animal and the percentage of successful trials in each group were assessed. The trained and not trained animals were sacrificed on the same day.

Prior to euthanasia, rats were weighed, temperature of the body nucleus was measured, and muscle endurance (time of hanging on a metal grid with the mesh size 1.5 × 1.5 mm, an average of three attempts) was evaluated. Prior to euthanasia, the rats received light inhalation anesthesia. Blood was collected from femoral vein to prepare blood plasma.

### Tissue homogenates

Immediately after euthanasia, the brain was harvested to isolate the olfactory bulb, cerebellum, pons + medulla oblongata, frontal lobe cortex, mesencephalon, and thalamencephalon. Samples (up to 150 mg in weight) of the isolated brain parts were weighed, placed in 1 mole of cold normal saline solution, and stored at a temperature of −20°C prior to assay (Vincek et al., 2003). Blood plasma samples were also stored at a temperature of −20°C. At the day of assay, the tissue samples were defrosted, brought to a concentration of 50 mg tissue/1 mL with normal saline, and homogenized. The homogenates were centrifuged for 25 min at 8000 g. The resulting supernatants were used to determine antioxidants and oxidants in each studied tissue. The assay was performed by a pairwise method with the same samples from young adult and aged rats.

### Chemiluminescence analysis of oxidants and antioxidants

The study was carried out on a Lumat LB 9507 (Berthold Technologies) two-flask high-resolution chemiluminometer with spectral sensitivity in the wavelength range of 390–620 nm. The intensity of chemiluminescence (CL) was recorded in relative light units (RLU). RLU = [(the measured number of pulses)/10] × stabilization factor of photomultiplier cathode sensitivity. The intensity of luminol-dependent CL was measured for 5 min.

#### Antioxidants

Antioxidant capacity of the tissue samples was determined by the modified method (Muller et al., 2012) where a source of radicals—the conjugate of Horseradish peroxidase with goat anti-mouse immunoglobulin—was replaced by the radicals produced by the Fenton reaction at the alkaline pH. In the radical-producing system, 1 mL H_2_O contains 30 μL of a 0.01 M luminol solution in a phosphate buffer pH 8.5, 20 μL of a 0.05 M FeSO_4_ solution, and 10 μL of 0.1 M H_2_O_2_. 1 mL of the radical-producing system was introduced into each of two Lumat LB 9507 flasks, the first flask was then supplemented with 10 μL H_2_O, and the second flask with 10 μL of supernatant of the test sample. The CL intensity was measured in the first and second flasks during 5 min. The difference between CL intensities of the radical-producing system (1st flask) and the radical-producing system supplemented with supernatant of the test sample (2nd flask) was used to plot a CL intensity curve characterizing the antioxidant capacity (AC). The light sum (Sm) of CL AC was found as the area under the curve using the Microsoft Excel program. The dimensions of Sm CL AC were presented as units/g tissue, where units = RLU·10^9^.

#### Oxidants

Oxidants (reactive oxygen species, ROS) were determined from the activated luminol chemiluminescence under the same CL conditions. 1 mL H_2_O of the system for ROS measurements contains 30 μL of a 0.01 M luminol solution in a phosphate buffer pH 8.5. mL of the system was placed into a Lumat LB 9507 flask and supplemented with 10 μL of supernatant of the test sample; CL intensity was measured for 5 min. After plotting the CL intensity curve for ROS characterization, Sm CL ROS was found as the area under the curve using the Microsoft Excel program. The dimensions of Sm CL ROS were presented as units/g tissue, where units = RLU·10^9^.

### Statistical analysis

Statistical analysis was carried out using the Statistica 6.0 software package for non-parametric samples. The data were expressed as the mean value ± SD. The groups were compared using the non-parametric Mann–Whitney test. The *p*-values of < 0.05 were considered to be significant. The Microsoft Excel program was used for processing the CL intensity curves and preparing the documents.

## Results

### Characteristics of 3-month-old and 11-month-old rats

General characteristics of the test animals are listed in Table 1.

**Table 1:** **Body weight, temperature of the body nucleus and muscle endurance of trained and not trained 3-month-old and 11-month-old rats**.

**Characteristic**	**Time of examination**			
	**One week prior to euthanasia**	**Before euthanasia**	**Before MWM**	**After MWM**
	**Not trained 3-month-old rats**		**Three-month-old rats trained in MWM**	
Body weight (g)	278.4 ± 9.1	311.5 ± 10.7	280.9 ± 4.4	306.6 ± 6.1
Body temperature (°C)	38.0 ± 0.1	37.1 ± 0.1	38.4 ± 0.1	37.2 ± 0.1
Muscle endurance (time of hanging on a metal grid) (s)	5.78 ± 1.67	9.50 ± 2.50	4.70 ± 0.83	11.60 ± 3.00^*^
	**Not trained 11-month-old rats**		**Eleven-month-old rats trained in MWM**	
Body weight (g)	576.0 ± 21.4	571.4 ± 22.6	600.9 ± 16.6	593.5 ± 17.4
Body temperature (°C)	37.4 ± 0.1	37.1 ± 0.1	37.8 ± 0.1	37.4 ± 0.2
Muscle endurance (time of hanging on a metal grid) (s)	1.10 ± 0.05	1.25 ± 0.17	1.07 ± 0.04	1.70 ± 0.23^*^

* p < 0.05 (the differences between groups of trained and not trained rats).

Physiological state of each animal corresponded to its age. After MWM training, 3-month-old and 11-month-old rats showed an increase in muscle endurance in comparison with the values obtained before training.

### Training in the Morris water maze

**Figure 1:**
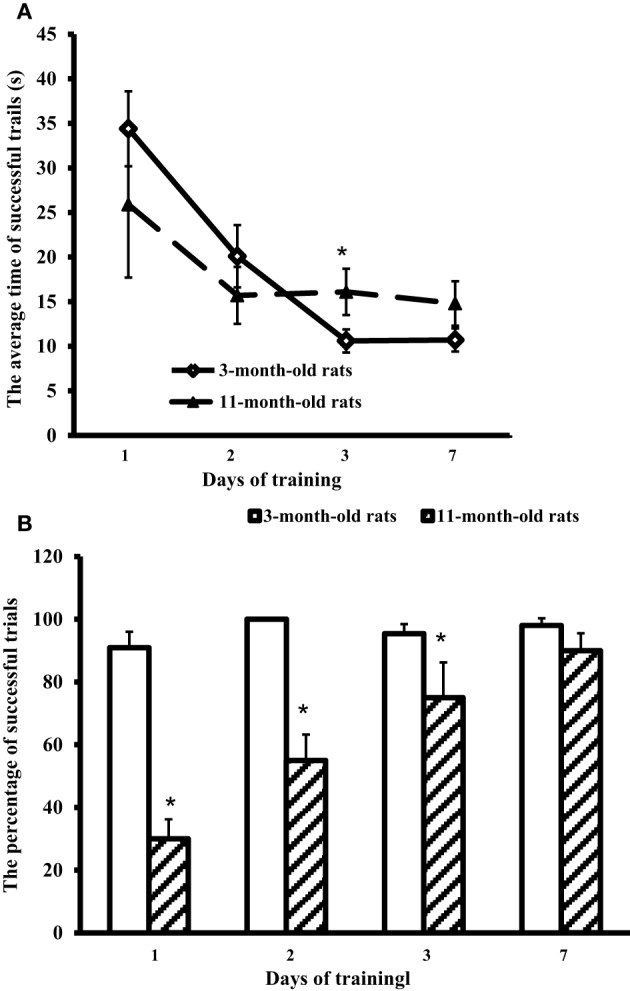
**Results of the Morris water-maze training. (A)** The average time of successful trials (s) and **(B)** the percentage of successful trials in 3-month-old and 11-month-old rats. ^*^p < 0.05 (the differences between groups of 3-month-old rats and 11-month-old rats).

All the rats were allowed swimming in the pool for 60 s. The rats were placed in the pool by hand in the segment opposite to the hidden platform. Young adult rats immediately started swimming along the pool walls, whereas aged rats first sank and started to swim only after several attempts. Trials were considered successful if the rat could find the hidden platform for 60 s. The average time of successful trials for each test day and the percentage of successful trials in each group were recorded (Figure 1).

Figure 1 shows that at the first training day 11-month-old rats had only ~30% of successful trials (against 90% in 3-month-old rats). However, on day 7 the scores of aged rats approached those of young adult rats (respectively, 90 and 98%, the difference is insignificant). The trial strategy used by 11-month-old rats differed from the strategy of young adult animals. In a successful trial, aged rats after the immersion sprawled on the water, looked around the pool walls, found the landmarks, and swam through the center of the pool directly to the hidden platform. So, the average time of successful trials in 11-month-old rats was 25.9 ± 8.2 at the first day, and on day 7 showed no significant difference from the first day, being equal to 14.8 ± 2.5 s (Figure 1A). On the contrary, young adult rats rapidly swam along the walls to find the hidden platform. Their average time of successful trials at the first day was 34.4 ± 4.2 s, significantly decreasing in the next days: day 2, 1 ± 3.5 s; day 3, 10.6 ± 1.3 s, and day 7, 10.7 ± 1.3 s (Figure 1A).

### Effect of the Morris water-maze training on antioxidants and oxidants in brain parts and blood plasma of 3-month-old rats

**Figure 2:**
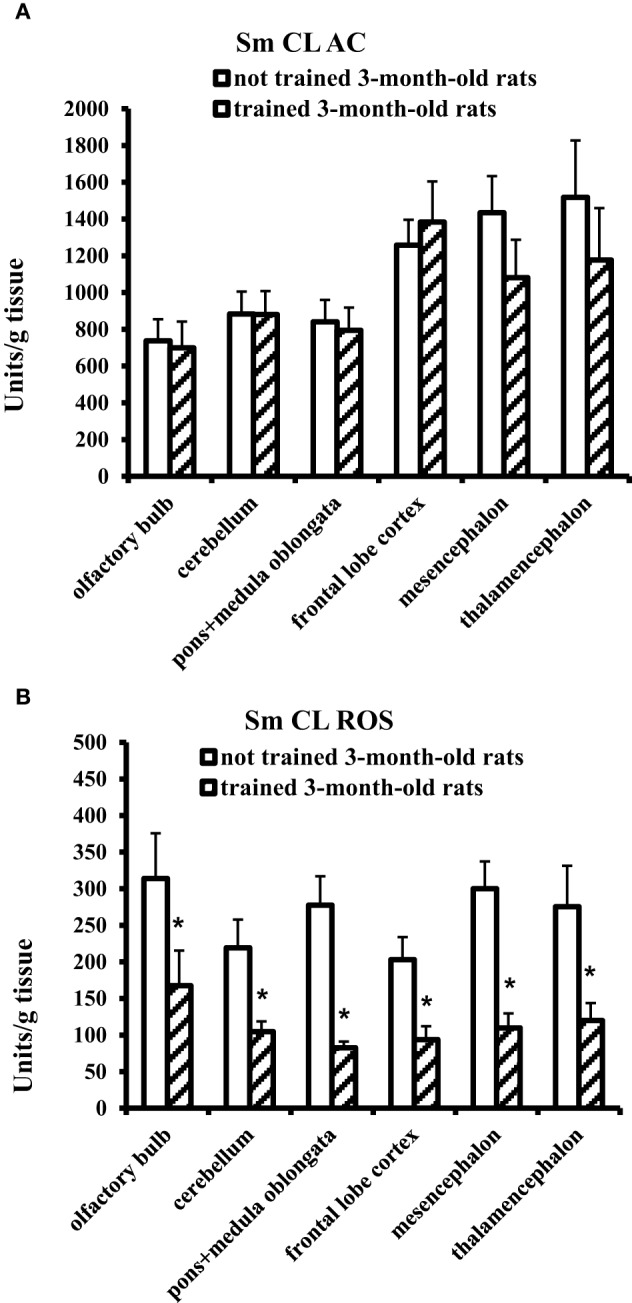
**The light sums of chemiluminescence for antioxidants (A Sm CL AC) and oxidants (B Sm CL ROS) in the brain part of 3-month-old rats after training in Morris water maze (“not trained 3-month-old rats,” *n* = 10; “trained 3-month-old rats,” *n* = 10)**. Brain parts: olfactory bulb, cerebellum, pons + medulla oblongata, frontal lobe cortex, mesencephalon, thalamencephalon. Along the Y axis, units = RLU·10^9^ (RLU is the relative light units). Data are presented as the mean value ± SD. ^*^p < 0.05 (the differences between groups of “not trained 3-month-old rats” and “trained 3-month-old rats”).

In 3-month-old young adult rats, the Morris water-maze training exerted no effect on **Sm CL AC** but significantly decreased **Sm CL ROS** in all the tested brain parts in comparison with not trained young adult rats (Figure 2).

**Figure 3:**
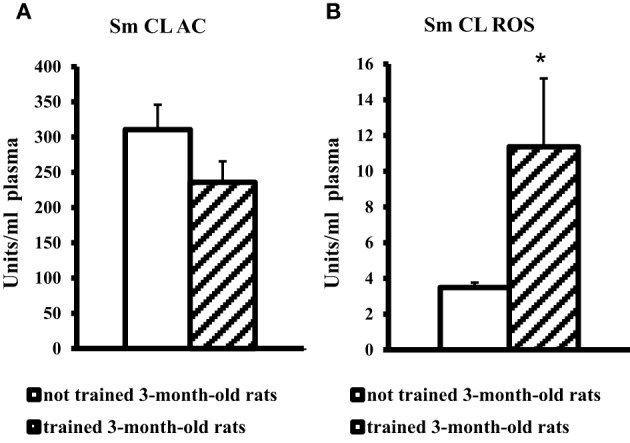
**The light sums of chemiluminescence for antioxidants (A Sm CL AC) and oxidants (B SM CL ROS) in blood plasma of 3-month-old rats after MWM training (“not trained 3-month-old rats,” *n* = 10; “trained 3-month-old rats,” *n* = 10)**. Along the Y axis, units = RLU·10^9^ (RLU is the relative light units). Data are presented as the mean value ± SD. ^*^p < 0.05 (the differences between groups of “not trained 3-month-old rats” and “trained 3-month-old rats”).

On the contrary, blood plasma of young adult rats after MWM training showed a significant, more than threefold increase in SM CL ROS and no changes in Sm CL AC as compared to not trained young adult rats (Figure 3).

### Effect of the Morris water-maze training on antioxidants and oxidants in brain parts and blood plasma of 11-month-old rats

**Figure 4:**
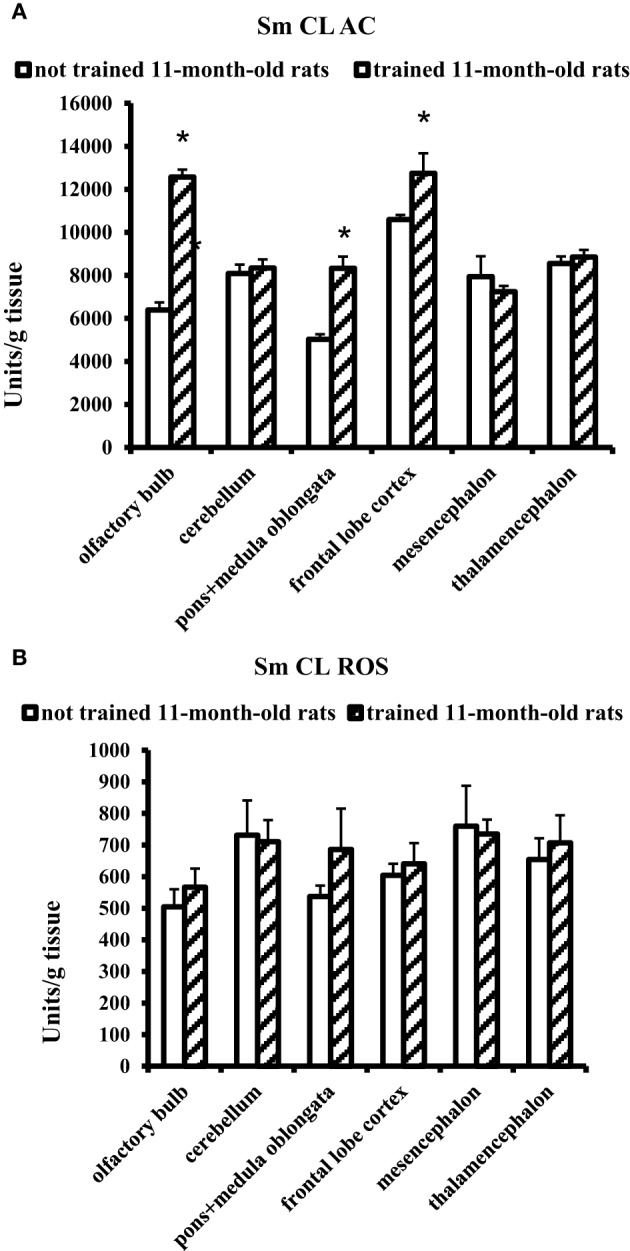
**The light sums of chemiluminescence for antioxidants (A Sm CL AC) and oxidants (B SM CL ROS) in the brain parts of 11-month-old rats after MWM training (“not trained 11-month-old rats,” *n* = 10; “trained 11-month-old rats,” *n* = 10)**. Brain parts: olfactory bulb, cerebellum, pons + medulla oblongata, frontal lobe cortex, mesencephalon, thalamencephalon. Along the Y axis, units = RLU·10^9^ (RLU is the relative light units). Data are presented as the mean value ± SD. ^*^p < 0.05 (the differences between groups of “not trained 11-month-old rats” and “trained 11-month-old rats”).

The effect of MWM training on 11-month-old rats was contrary to the effect obtained for young adult rats. In 11-month-old aged rats, a significant increase in Sm CL AC was observed in three brain parts: olfactory bulb, pons + medulla oblongata, and frontal lobe cortex. Sm CL ROS in trained aged rats did not change as compared to not trained aged rats (Figure 4).

**Figure 5:**
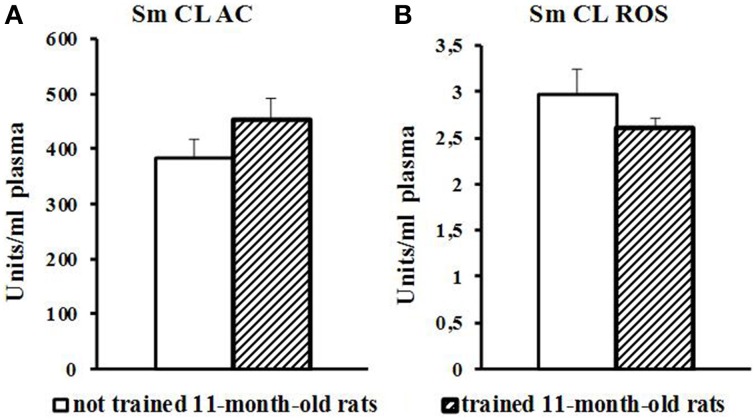
**The light sums of chemiluminescence for antioxidants (A Sm CL AC) and oxidants (B SM CL ROS) in blood plasma of 11-month-old rats after MWM training (“not trained 11-month-old rats,” *n* = 10; “trained 11-month-old rats,” *n* = 10)**. Along the Y axis, units = RLU·10^9^ (RLU is the relative light units). Data are presented as the mean value ± SD. *p* < 0.05 (the differences between groups of “not trained 11-month-old rats” and “trained11-month-old rats”).

Blood plasma of trained 11-month-old aged rats showed no changes in Sm CL AC and Sm CL ROS as compared to not trained 11-month-old aged rats (Figure 5).

## Discussion

Our study has demonstrated that not trained aged male Wistar rats have higher values of Sm CL ROS (oxidants) and Sm CL AC (antioxidants) as compared to not trained young adult male Wistar rats (a comparison of “not trained young adult rats” and “not trained aged rats” is illustrated in Figures 2, 4). This conclusion agrees with the data indicating that oxidative stress in the brain tissue of rodents starts to develop already in the middle age. An increase in the markers of ROS production and markers of oxidative damage was found in the brain parts of 12-month-old Male Brown Norway (BN) rats (Kodavanti et al., 2011) along with manifestation of selective neuronal vulnerability to oxidative stress (Wang and Michaelis, 2010). This could be a reason why in the first days of MWM training the aged rats showed a reliable “lag” from young adult rats, although on day 7 animals from both groups had almost equal results (Figure 1). However, the oxidative stress is usually accompanied by a decrease in activity of antioxidant systems; for example, the activity of antioxidant enzymes in brain was shown to decline upon aging (Sandhu and Kaur, 2002). Our study has revealed high levels of antioxidants (Sm CL AC) in the tissues of all the tested brain parts taken from not trained aged rats in comparison with not trained young adult rats. A possible reason is that our study determined non-enzymatic AC, i.e., the direct ability of the test sample to quench chemiluminescence of the radical producing system. In this case, the AC could be caused by oxidatively modified macromolecules, as it was reported, for example, in Dröge (2002).

It was found that MWM training exerts an effect on the redox processes that occur in the brain of young adult and aged male rats. However, this effect manifested itself in opposite directions of the redox processes: trained young adult rats showed a decrease in prooxidant activity (Sm CL ROS) in all the tested brain parts as compared to not trained young adult rats, whereas trained aged rats demonstrated an increase in antioxidant activity (Sm CL AC) in three brain parts as compared to not trained aged rats. This difference could be related to different age of the animals and/or to different behavioral strategies in MWM task. The trained young adult rats carried out the MWM task with a considerable physical activity: they intensely swam along the pool walls to search for the hidden platform and very seldom crossed the center of the pool (even in the last days of training), which is due to their instinctive behavior.

The ratio of redox processes in blood plasma of trained young adult rats differed from that in the brain: Sm CL AC did not change, while Sm CL ROS increased nearly threefold in comparison with not trained young adult rats (Figure 3). The increase in prooxidant activity of blood plasma after MWM training could be caused by physical activity and changes in the oxygen metabolism in muscular tissue (Towse et al., 2005).

In 11-month-old aged rats, the performance of MWM task was hindered not only by cognitive decline and mnemonic dysfunction (Bizon et al., 2009) but also by muscle weakness; so, they swam less than trained young adult rats and spent much time for spatial orientation. Blood plasma of trained aged rats showed no difference from not trained aged rats (Figure 5).

For trained 11-month-old aged rats, an increase in Sm CL AC was observed only in three of the tested brain parts: olfactory bulb, pons + medulla oblongata and frontal lobe cortex (Figure 4). In other parts of the brain, including thalamic area, no changes in redox processes were found in the experiments covering thalamus and adjacent areas.

It should be noted that the increase in Sm CL AC serves here as a non-enzymatic mechanism of restricting the oxidative stress in the brain upon aging.

Specific low- and macromolecular redox mechanisms were not elucidated in this work because AC is determined not only by the number of such molecules but also by their interactions. In addition, individual macromolecules often exhibit both pro- and AC (Dröge, 2002).

A successful performance of MWM task depends on the concerted action of different regions of the brain (hippocampus, striate body, basal parts of neoencephalon, cerebellum and cerebral cortex) and neurotransmitter systems constituting a functionally integrated neural network (O'Keefe and Burgess, 1996; D'Hooge and De Deyn, 2001; Hafting et al., 2005; Griffith et al., 2014). Some disturbances of redox mechanisms leading to oxidative stress were found in the structures of the aging brain (Gemma et al., 2007) as well as a selective neuronal vulnerability to oxidative stress (Wang and Michaelis, 2010), which could affect the activity of pro- and antioxidant processes in different parts of the brain upon aging.

It seems important that our study has demonstrated the effect of MWM procedure on non-enzymatic AC of some brain parts in 11-month-old rats because this is one of the mechanisms restricting the oxidative stress. Note that the health benefits of training, in particular upon aging, are evident but the mechanisms of implementing such benefits are not completely clear.

Among other factors that should be taken into account for estimation of MWM performance are the immersion stress, fatigue and sensory-motor deficits (Vorhees and Williams, 2006; Foster, 2012; Guidi and Foster, 2012). Motor deficits in aged animals could also deteriorate MWM performance in the experiments. However, the effect of additional physical activity during the MWM training on cognitive functions could facilitate learning of aged rats (Hillman et al., 2008).

The changes in redox processes that were revealed in the brain of young adult and aged rats after MWM may persist for a certain time; this may be one of the mechanisms providing long-term memories of the water maze (van Groen et al., 2002) and involved in carryover effects at further learning sessions (Guidi et al., 2014).

## Conclusion

Thus, our study has demonstrated a decrease in the initial performance in MWM in 11-month-old rats and their ability to successful learning after MWM. It was shown that the MWM procedure *per se* produces changes in different processes of redox homeostasis in 11-month-old and 3-month-old rats. Both these processes (a decrease in Sm CL ROS in young rats and an increase in Sm CL AC in 11-month-old rats) are favorable in terms of the oxidative stress restriction. It is commonly accepted that training improves health, particularly upon aging; however, the mechanisms of this effect need further elucidation. Thus, in-depth investigation of the effect produced by physical training or MWM on redox homeostasis is necessary in order to reveal all aspects of the oxidative damage and possible mechanism of its reparation. Results of the study may be an argument for further development of the animal training procedures aimed to activate the mechanisms that can prevent the age-related deterioration of performance in the learning test. This may be useful not only for devising the training procedures applicable to human patients with age-related cognitive deficits but also for their rehabilitation, non-drug therapy of such deficits and creation of additional motivation for a healthy lifestyle.

### Conflict of interest statement

The authors declare that the research was conducted in the absence of any commercial or financial relationships that could be construed as a potential conflict of interest.
